# Association between Bisphenol A exposure and body composition parameters in children

**DOI:** 10.3389/fendo.2023.1180505

**Published:** 2023-05-18

**Authors:** Yong Guo, Cui Liu, Yu-Hong Deng, Jing Ning, Li Yu, Jie-Ling Wu

**Affiliations:** Department of Children’s Health Care, Guangdong Women and Children Hospital, Guangzhou Medical University, Guangzhou, China

**Keywords:** Bisphenol (BPA), body composition parameter, children, obesity, bioelectrical impedance analysis

## Abstract

**Background:**

Although there is evidence linking Bisphenol A (BPA) exposure to obesity, research examining its relationship with body composition parameters in young children is limited.

**Methods:**

A cross-sectional investigation was conducted on 200 preschool children aged between 4 and 6 years in Guangzhou, China. BPA exposure was assessed through urine samples using ultra-high performance liquid chromatography- tandem mass spectrometry, and body composition parameters were measured through bioelectrical impedance analysis (InBody770).

**Results:**

The median urinary BPA concentration was 0.556 μg/L (IQR: 0.301 - 1.031 μg/L) and creatinine-adjusted BPA concentration was 0.930 μg/g (IQR: 0.551 - 1.586 μg/g). BPA levels were significantly associated with body mass index (β= 1.15; 95%CI: 0.47, 1.83), body fat mass (β= 1.14; 95%CI: 0.39, 1.89), fat free mass (β= 0.92; 95%CI: 0.26, 1.58), and percent body fat (β= 3.44; 95%CI: 1.17, 5.71) after adjusting for potential confounding factors. Similarly, adjusted models with log_10_-transformed creatinine-adjusted BPA concentrations as a continuous variable showed similar trends. Positive linear associations were observed between quartiles of BPA concentrations and body composition parameters, with the highest coefficients in the fourth quartile.

**Conclusion:**

Our study provides further evidence of positive correlations between BPA exposure and body composition parameters in children aged 4 to 6 years. These findings highlight the potential health risks associated with obesity-related body composition parameters in young children. Further investigations are needed to confirm this association and explore the underlying mechanisms.

## Introduction

Bisphenol A (BPA), a chemical compound, finds broad application in the manufacturing of polycarbonate plastics and epoxy resins (1). BPA exposure has been associated with multiple adverse health effects, such as obesity, insulin resistance, cardiovascular disease, reproductive disorders, and neurobehavioral disorders (2–4). Infants and young children may be exposed to BPA through various pathways, including the utilization of polycarbonate feeding bottles and drinking cups, canned provisions, thermal paper goods, and maternal exposure during pregnancy and breastfeeding (5–7). In addition, exposure to BPA may also occur through dust particles in indoor environments and personal care products such as shampoos, lotions, and cleaning products (8, 9). There is growing concern regarding BPA exposure during critical early developmental periods, as it can disrupt hormonal regulation and cause long-lasting effects. Numerous studies have explored the potential link between BPA exposure and childhood obesity, potentially due to its ability to mimic estrogen and disrupt normal hormonal balance, interfere with insulin signaling, stimulate the differentiation of preadipocytes, alter the gut microbiome, induce inflammation, and affect energy metabolism (10–13). Recent research has also suggested that BPA exposure may contribute to childhood obesity by influencing epigenetic modifications of genes involved in metabolism and energy balance (14, 15). These findings highlight the potential for environmental factors such as BPA exposure to contribute to the obesity epidemic, and underscore the importance of reducing exposure to BPA, particularly in children. Although there is growing research on the relationship between BPA exposure and childhood obesity, studies on the relationship between BPA exposure and body composition parameters remain scarce. Understanding the impact of BPA on body composition parameters is critical for a better understanding of the link between BPA and childhood obesity, as well as for implementing more effective measures to prevent childhood obesity. Additionally, as body composition parameters are closely related to the risk of cardiovascular disease, metabolic disorders, and other health issues, investigating the relationship between BPA exposure and body composition parameters can help shed light on the health effects of BPA and its related mechanisms (16–18). Therefore, further investigations are required to examine the association between BPA exposure and body composition parameters to further understand the potential harm. The purpose of this study is to investigate the potential correlation between exposure to BPA and alterations in body composition parameters, with the intention of elucidating the potential impact of BPA on the development of obesity and related health issues.

## Materials and methods

### Study population

The cross-sectional study was conducted on a sample of 200 preschool children aged 4 to 6 years in Guangzhou, China, during the period between September and November of 2020. Specifically, we selected one preschool and used a stratified sampling method to randomly select three classes from each age group. Parents were then informed of the study and gave their consent for their child to participate. Inclusion criteria: participants must be older than 3 years of age, have a height greater than 95 cm, and weight more than 10 kg. Children with implanted cardiac pacemakers or other electronic devices, as well as those with limb deficiencies that prevented adequate electrode contact, were excluded from the study. Information on the children’s sociodemographic characteristics and health status was gathered by structured questionnaires.

### Ethical statement

This study was approved by the Medical Research Ethics Board of Guangdong Women and Children Hospital. Participants were fully informed of the study procedures and objectives, and informed written parental consent was obtained prior to enrollment in the study.

### BPA exposure assessment

Urine samples were collected from participants using 10 ml polypropylene tubes and stored at -20°C for subsequent BPA concentration analysis. BPA was detected using ultra-high performance liquid chromatography- tandem mass spectrometry, following established protocols (19) and prior studies (20, 21). Briefly, 4 mL of urine was mixed with 0.50 mL of phosphate buffer (pH=7.0) and 40 μL of β-glucuronidase, and underwent hydrolysis. The hydrolyzed urine samples were extracted twice with ethyl acetate:n-hexane (1:1) and the upper organic phase was dissolved in 40% acetonitrile-water solution for analysis. BPA concentration was calibrated against creatinine, which was measured using an automatic biochemical instrument. BPA concentrations in the 25th, 50th, and 75th percentiles were utilized as dividing points, given the potential non-monotonic dose response effects of BPA (22). Urine samples were processed and stored under controlled conditions, with background error from laboratory vessels being below the instrument’s detection limit. The precision recoveries of BPA in urine were within acceptable limits, with inter-batch and intra-batch precision recoveries ranging from 92.6%-120.0% and 3.54%-7.02%, respectively.

### Measurements of body composition

All study participants underwent anthropometric measurements. Participants’ heights were measured to the nearest 0.1 cm, and weights were measured to the nearest 0.1 kg, with minimal clothing. The body composition was assessed via bioelectrical impedance analysis, employing the InBody770 device. The test body composition parameters included body mass index (BMI), body fat mass (BFM), soft lean mass (SLM), fat free mass (FFM), skeletal muscle mass (SMM), percent body fat (PBF), waist-hip ratio (WHR), visceral fat area (VFA), arm circumference (AC), arm muscle circumference (AMC), fat free mass index (FFMI), fat mass index (FMI) and skeletal muscle index (SMI). The age and sex standardized BMI z-score was calculated based on the reference of Chinese children under 7 years in 2015 (23). Children were categorized as non-overweight (BMI z-score ≤ 1) and overweight/obesity (BMI z-score > 1) according to the BMI z-score.

### Statistical analyses

The Kolmogorov-Smirnov test was utilized to assess the normal distribution of data presented as mean ± standard deviation (SD), while non-normally distributed data were expressed as medians (quartiles). For BPA concentrations (μg/L), the median value and interquartile range (IQR) were determined. Urinary BPA concentrations were measured in both volume-based (μg/L) and creatinine-adjusted formats (μg/g) to assess the level of exposure to BPA in the participants (24), and log_10_-transformation was performed to account for non-normal distribution. The Pearson correlation coefficient was used to analyze the relationship between log-transformed BPA concentrations and body composition parameters. We utilized linear regression models to assess the relationship between body composition parameters and BPA concentrations. Beta coefficients indicated the average change in body composition parameters per unit increase in log-transformed BPA concentrations, or the mean variation in body composition parameters between the first quartile of BPA exposure and subsequent three quartiles, together with corresponding 95% Confidence Intervals (CIs). The models were adjusted for children’s age, sex, primary caregiver, caregiver education, gestational age at birth, birth weight, and duration of breastfeeding. Statistical analyses were performed using R software version 4.2.1 (www.R-project.org) and the SPSS statistical software package (V26, IBM Statistics, Chicago, IL, USA). Statistical significance was set at P <0.05 for all analyses.

## Results

Of the 200 children aged 4 to 6 years who enrolled in our study, 120 were male. The mean (± SD) age of the children was 5.22 ( ± 0.67) years. 76.0% of participants reported the primary caregivers were mothers. BPA exposure was detected in all urine samples. The median (IQR) urinary BPA concentrations and creatinine-adjusted BPA concentrations were 0.556 μg/L (0.301 - 1.031 μg/L) and 0.930 μg/g (0.551 - 1.586 μg/g), respectively. The other demographic characteristics and measurement indicators are presented in **Table 1**.

**Table 1:** Characteristics and measurement indicators of the participants.

Characteristics	Total sample (n=200)	Male (n=120)	Female (n=80)
Age, years, mean ± SD	5.22 ± 0.67	5.24 ± 0.69	5.19 ± 0.65
Primary caregiver, n(%)			
Father	20 (10.0)	11 (9.2)	9 (11.3)
Mother	152 (76.0)	92 (76.7)	60 (75.0)
Others	28 (14.0)	17 (14.2)	11 (13.8)
Caregiver education, n(%)			
Junior high school or below	81 (40.5)	52 (43.3)	29 (36.3)
Senior high school	66 (33.0)	39 (32.5)	27 (33.8)
College or above	53 (26.5)	29 (24.2)	24 (30.0)
Gestational age at birth, weeks, mean ± SD	39.01 ± 1.25	38.88 ± 1.21	39.20 ± 1.28
Birth weight, kg, mean ± SD	3.21 ± 0.37	3.22 ± 0.40	3.18 ± 0.34
Duration of breastfeeding, months, median(25th-75th)	8.0 (4.5 - 11.0)	7.0 (4.0 - 11.0)	9.0 (5.0 - 12.0)
BPA levels, μg/L, median(25th-75th)	0.556 (0.301 - 1.031)	0.629 (0.325 - 1.148)	0.457 (0.290 - 0.880)
Creatinine-adjusted BPA levels, μg/g, median(25th-75th)	0.930 (0.551 - 1.586)	0.930 (0.548 - 1.647)	0.950 (0.579 - 1.493)
Body mass index, kg/m^2^, mean ± SD	15.46 ± 1.81	15.69 ± 1.99	15.11 ± 1.44
Body mass index z score	-0.03 ± 1.09	0.02 ± 1.20	-0.09 ± 0.89
Body fat mass, kg, mean ± SD	3.46 ± 1.97	3.50 ± 2.24	3.41 ± 1.51
Soft lean mass, kg, mean ± SD	14.75 ± 2.25	15.26 ± 2.35	13.98 ± 1.85
Fat free mass, kg, mean ± SD	15.65 ± 2.38	16.18 ± 2.49	14.87 ± 1.98
Skeletal muscle mass, kg, mean ± SD	7.25 ± 1.42	7.58 ± 1.48	6.77 ± 1.16
Percent body fat, %, mean ± SD	17.33 ± 5.92	16.76 ± 6.38	18.19 ± 5.08
Waist-hip ratio, mean ± SD	0.75 ± 0.03	0.75 ± 0.03	0.75 ± 0.03
Visceral fat area, cm^2^, mean ± SD	15.47 ± 7.77	15.82 ± 9.04	14.93 ± 5.34
Arm circumference, cm, mean ± SD	18.72 ± 1.93	18.97 ± 2.12	18.33 ± 1.55
Arm muscle circumference, cm, mean ± SD	15.01 ± 1.59	15.27 ± 1.73	14.62 ± 1.27
Fat free mass index, mean ± SD	12.68 ± 0.77	12.94 ± 0.75	12.30 ± 0.64
Fat mass index, mean ± SD	2.77 ± 1.34	2.75 ± 1.49	2.81 ± 1.07
Skeletal muscle index, mean ± SD	3.81 ± 0.62	4.00 ± 0.61	3.52 ± 0.53

**Figure 1:**
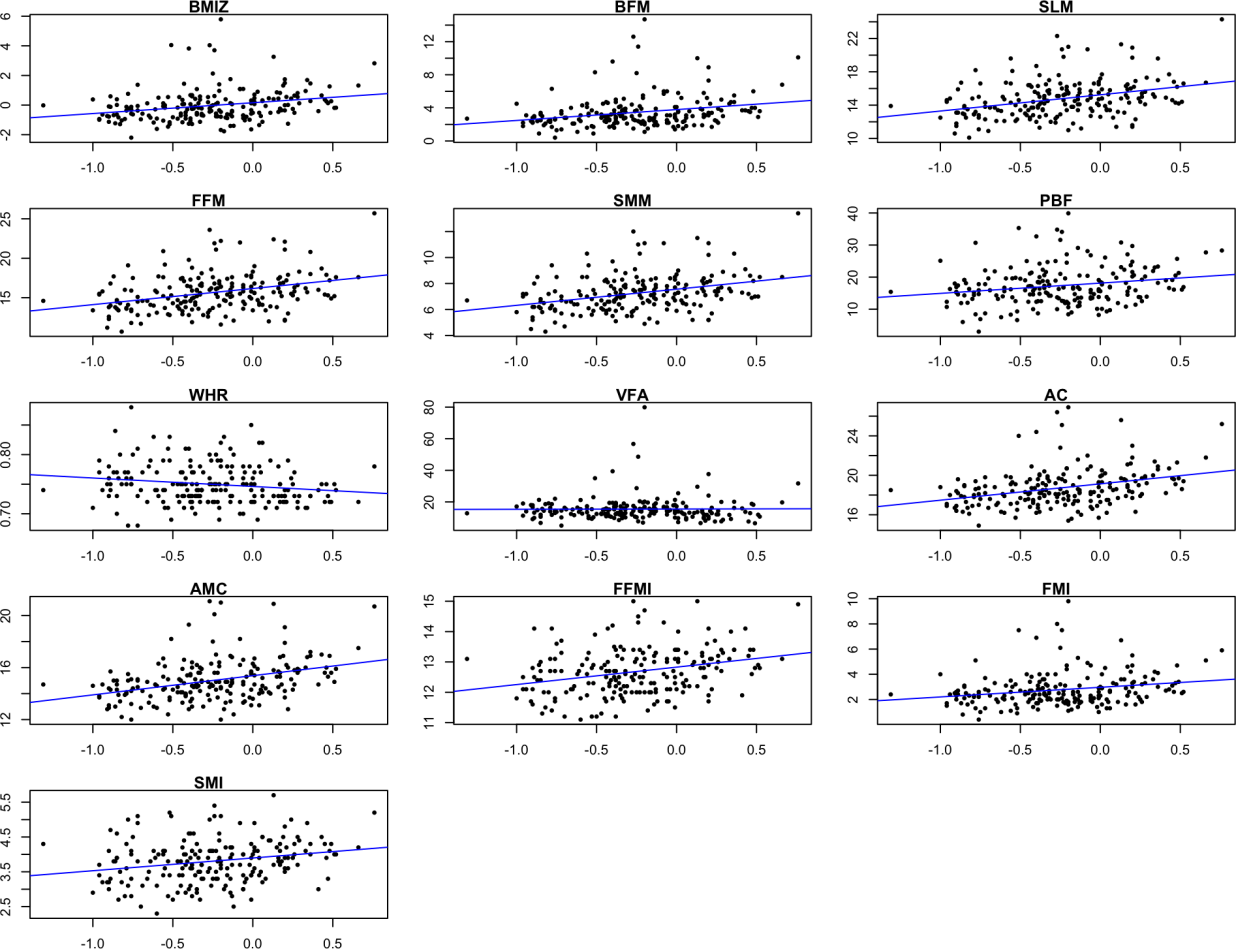
Scatter plots showing the correlation between log-transformed BPA levels and body composition parameters. BMIZ, body mass index z score; BFM, body fat mass; SLM, soft lean mass; FFM, fat free mass; SMM, skeletal muscle mass; PBF, percent body fat; WHR, waist-hip ratio; VFA, visceral fat area; AC, arm circumference; AMC, arm muscle circumference; FFMI, fat free mass index; FMI, fat mass index; SMI, skeletal muscle index.

**Figure 2:**
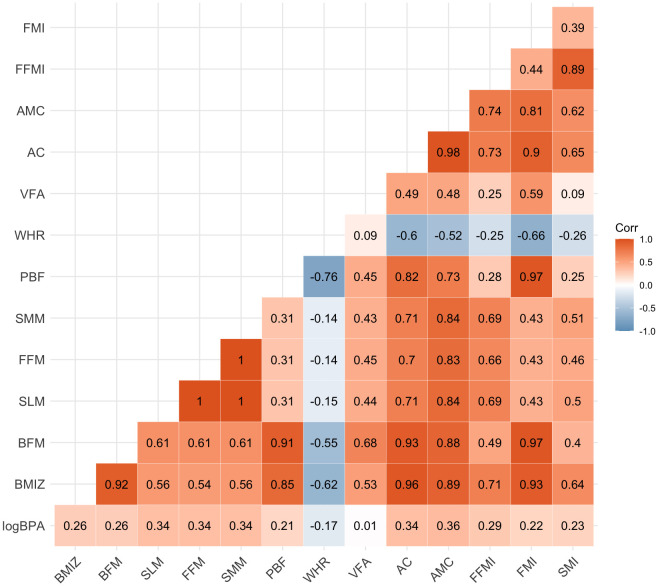
Heat map on the correlation between log-transformed BPA levels and body composition parameters. Values represent Pearson’s correlation coefficient. BPA, bisphenol A; BMIZ, body mass index z score; BFM, body fat mass; SLM, soft lean mass; FFM, fat free mass; SMM, skeletal muscle mass; PBF, percent body fat; WHR, waist-hip ratio; VFA, visceral fat area; AC, arm circumference; AMC, arm muscle circumference; FFMI, fat free mass index; FMI, fat mass index; SMI, skeletal muscle index.

**Figures 1**, **2** showed the correlation between log-transformed BPA levels and body composition parameters. Log_10_-transformed BPA levels were found to have positive correlations with BMI, BPF, SLM, FFM, SMM, PBF, AC, AMC, FFMI, FMI and SMI. In general, higher BPA levels were found to be associated with higher body composition parameters. The adjusted models revealed significant correlations between elevated BPA concentrations and increased BMI (β= 1.15; 95%CI: 0.47, 1.83), BFM (β= 1.14; 95%CI: 0.39, 1.89), FFM (β= 0.92; 95%CI: 0.26, 1.58), and PBF (β= 3.44; 95%CI: 1.17, 5.71). Similarly, adjusted models with log_10_-transformed creatinine-adjusted BPA concentrations as a continuous variable showed similar trends in the results, as presented in **Table 2**.

**Table 2:** Association of BPA levels and body composition parameters.

Body composition parameters	log-transformed BPA (β [95% CI])		log-transformed BPA/Creatinine (β [95% CI])	
	Crude	Adjusted	Crude	Adjusted
Body mass index	1.33 (0.71, 1.95)	1.15 (0.47, 1.83)	1.20 (0.55, 1.85)	1.14 (0.46, 1.82)
Body mass index z score	0.73 (0.35, 1.11)	0.67 (0.25, 1.08)	0.71 (0.32, 1.10)	0.67 (0.25, 1.09)
Body fat mass	1.32 (0.63, 2.00)	1.14 (0.39, 1.89)	1.10 (0.38, 1.82)	1.11 (0.36, 1.86)
Soft lean mass	1.95 (1.19, 2.71)	0.90 (0.27, 1.52)	0.91 (0.08, 1.73)	0.75 (0.12, 1.38)
Fat free mass	2.05 (1.25, 2.85)	0.92 (0.26, 1.58)	0.93 (0.05, 1.81)	0.76 (0.10, 1.43)
Skeletal muscle mass	1.24 (0.77, 1.72)	0.58 (0.19, 0.97)	0.58 (0.06, 1.10)	0.49 (0.09, 0.88)
Percent body fat	3.22 (1.14, 5.29)	3.44 (1.17, 5.71)	3.44 (1.29, 5.59)	3.44 (1.16, 5.72)
Waist-hip ratio	-0.01 (-0.03, 0.00)	-0.01 (-0.03, 0.00)	-0.02 (-0.03, 0.00)	-0.01 (-0.03, 0.00)
Visceral fat area	0.18 (-2.61, 2.97)	0.01 (-3.03, 3.05)	-0.01 (-2.90, 2.88)	0.48 (-2.58, 3.53)
Arm circumference	1.66 (1.01, 2.32)	1.31 (0.61, 2.01)	1.33 (0.64, 2.03)	1.24 (0.53, 1.94)
Arm muscle circumference	1.48 (0.95, 2.01)	1.02 (0.48, 1.57)	1.03 (0.45, 1.60)	0.92 (0.37, 1.47)
Fat free mass index	0.58 (0.31, 0.84)	0.39 (0.12, 0.65)	0.44 (0.16, 0.72)	0.38 (0.11, 0.64)
Fat mass index	0.77 (0.30, 1.24)	0.77 (0.26, 1.29)	0.76 (0.28, 1.25)	0.77 (0.25, 1.29)
Skeletal muscle index	0.37 (0.15, 0.58)	0.28 (0.06, 0.50)	0.31 (0.08, 0.54)	0.28 (0.06, 0.50)

Adjusted for children’s age, sex, primary caregiver, caregiver education, gestational age at birth, birth weight, and duration of breastfeeding.

Our results revealed that BPA exposure was more strongly associated with increases in BMI, BFM, PBF, and FMI in males compared to females (**Table 3**). The linear regression analysis employing quartiles of BPA concentrations as predictor variables, with the first quartile used as the reference group, indicated positive linear associations between BPA concentrations and BMI, BPF, SLM, FFM, SMM, PBF, AC, AMC, FFMI, FMI, and SMI. The coefficients of these associations between BPA concentrations and body composition parameters were considerably greater in the fourth quartile of BPA exposure as compared to those in the first quartile. However, when quartiles of creatinine-adjusted BPA concentrations were considered, all these associations were attenuated, as shown in **Table 4**. There were 23 children identified as overweight/obesity. The results showed increased odds of overweight/obesity (OR = 4.81; 95% CI: 1.19, 19.49) among children with log-transformed BPA concentrations after controlling for confounders.

**Table 3:** Association of BPA levels and body composition parameters by sex.

Body composition parameters	log-transformed BPA (adjusted β [95% CI])		log-transformed BPA/Creatinine (adjusted β [95% CI])	
	Male	Female	Male	Female
Body mass index	1.52 (0.56, 2.48)	0.76 (-0.17, 1.70)	1.52 (0.59, 2.44)	0.43 (-0.60, 1.46)
Body mass index z score	0.88 (0.3, 1.46)	0.48 (-0.10, 1.06)	0.89 (0.33, 1.45)	0.26 (-0.38, 0.90)
Body fat mass	1.78 (0.72, 2.83)	0.30 (-0.67, 1.27)	1.56 (0.52, 2.59)	0.21 (-0.85, 1.28)
Soft lean mass	0.68 (-0.19, 1.55)	1.43 (0.57, 2.29)	0.56 (-0.28, 1.41)	1.19 (0.23, 2.16)
Fat free mass	0.71 (-0.21, 1.63)	1.47 (0.56, 2.38)	0.56 (-0.33, 1.45)	1.26 (0.24, 2.28)
Skeletal muscle mass	0.43 (-0.11, 0.98)	0.93 (0.39, 1.46)	0.36 (-0.17, 0.89)	0.77 (0.17, 1.38)
Percent body fat	6.09 (3.06, 9.11)	-0.55 (-3.97, 2.87)	5.47 (2.51, 8.42)	-0.97 (-4.70, 2.75)
Waist-hip ratio	-0.02 (-0.04, 0.00)	0.00 (-0.02, 0.02)	-0.02 (-0.04, -0.01)	0.01 (-0.01, 0.04)
Visceral fat area	1.40 (-3.12, 5.93)	-1.32 (-4.81, 2.17)	0.64 (-3.74, 5.02)	1.28 (-2.52, 5.09)
Arm circumference	1.71 (0.72, 2.69)	0.84 (-0.14, 1.81)	1.62 (0.66, 2.57)	0.44 (-0.64, 1.52)
Arm muscle circumference	1.26 (0.49, 2.02)	0.78 (0.04, 1.53)	1.13 (0.39, 1.87)	0.48 (-0.35, 1.31)
Fat free mass index	0.27 (-0.09, 0.63)	0.68 (0.28, 1.08)	0.37 (0.03, 0.72)	0.44 (-0.02, 0.90)
Fat mass index	1.28 (0.57, 2.00)	0.05 (-0.66, 0.77)	1.16 (0.46, 1.86)	-0.04 (-0.82, 0.74)
Skeletal muscle index	0.14 (-0.16, 0.44)	0.56 (0.22, 0.90)	0.27 (-0.02, 0.56)	0.30 (-0.10, 0.69)

Adjusted for children’s age, primary caregiver, caregiver education, gestational age at birth, birth weight, and duration of breastfeeding.

**Table 4:** Association of BPA quartiles and body composition parameters.

Body composition parameters	BPA quartiles (μg/L), adjusted β (95% CI)				BPA/creatinine quartiles (μg/g), adjusted β (95% CI)			
	1^st^ (0.049-0.301)	2^nd^ (0.302-0.556)	3^rd^ (0.557-1.031)	4^th^ (1.032-5.808)	1^st^ (0.84-0.551)	2^nd^ (0.552-0.930)	3^rd^ (0.931-1.586)	4^th^ (1.587-9.520)
Body mass index	Ref.	0.73 (0.64, 1.30)	0.03 (-0.08, 0.57)	1.43 (1.35, 2.03)	Ref.	0.42 (-0.3, 1.13)	0.43 (-0.30, 1.16)	0.71 (-0.02, 1.45)
Body mass index z score	Ref.	0.45 (0.03, 0.88)	0.38 (-0.06, 0.82)	0.78 (0.33, 1.22)	Ref.	0.25 (-0.18, 0.69)	0.25 (-0.20, 0.70)	0.42 (-0.03, 0.87)
Body fat mass	Ref.	0.79 (0.02, 1.56)	0.71 (-0.08, 1.50)	1.25 (0.44, 2.05)	Ref.	0.56 (-0.22, 1.35)	0.52 (-0.28, 1.32)	0.63 (-0.18, 1.43)
Soft lean mass	Ref.	0.45 (-0.20, 1.11)	0.46 (-0.20, 1.13)	0.76 (0.09, 1.44)	Ref.	0.11 (-0.54, 0.76)	0.15 (-0.52, 0.81)	0.58 (-0.09, 1.26)
Fat free mass	Ref.	0.48 (-0.21, 1.17)	0.50 (-0.20, 1.20)	0.77 (0.06, 1.48)	Ref.	0.12 (-0.57, 0.80)	0.16 (-0.55, 0.86)	0.60 (-0.11, 1.31)
Skeletal muscle mass	Ref.	0.28 (-0.13, 0.69)	0.28 (-0.14, 0.70)	0.50 (0.07, 0.92)	Ref.	0.07 (-0.34, 0.48)	0.11 (-0.31, 0.53)	0.38 (-0.04, 0.80)
Percent body fat	Ref.	2.45 (0.11, 4.79)	1.80 (-0.59, 4.18)	4.04 (1.61, 6.47)	Ref.	1.91 (-0.47, 4.29)	1.38 (-1.05, 3.81)	1.82 (-0.63, 4.27)
Waist-hip ratio	Ref.	-0.01 (-0.02, 0.00)	0.00 (-0.02, 0.01)	-0.02 (-0.03, 0.00)	Ref.	0.00 (-0.02, 0.01)	0.00 (-0.01, 0.01)	-0.01 (-0.02, 0.01)
Visceral fat area	Ref.	1.41 (-1.71, 4.53)	2.62 (-0.56, 5.80)	-0.17 (-3.41, 3.07)	Ref.	1.22 (-1.90, 4.34)	1.55 (-1.64, 4.74)	-0.47 (-3.69, 2.74)
Arm circumference	Ref.	0.83 (0.10, 1.55)	0.66 (-0.08, 1.40)	1.44 (0.69, 2.19)	Ref.	0.50 (-0.24, 1.25)	0.45 (-0.30, 1.21)	0.8 (0.04, 1.57)
Arm muscle circumference	Ref.	0.64 (0.08, 1.20)	0.52 (-0.06, 1.09)	1.09 (0.50, 1.67)	Ref.	0.38 (-0.19, 0.95)	0.32 (-0.26, 0.91)	0.63 (0.04, 1.22)
Fat free mass index	Ref.	0.17 (-0.10, 0.44)	0.17 (-0.11, 0.45)	0.41 (0.13, 0.69)	Ref.	0.00 (-0.28, 0.27)	0.08 (-0.2, 0.36)	0.29 (0.01, 0.57)
Fat mass index	Ref.	0.56 (0.03, 1.10)	0.48 (-0.07, 1.02)	0.90 (0.35, 1.45)	Ref.	0.42 (-0.12, 0.96)	0.35 (-0.20, 0.9)	0.42 (-0.14, 0.98)
Skeletal muscle index	Ref.	0.09 (-0.14, 0.31)	0.04 (-0.19, 0.27)	0.34 (0.11, 0.58)	Ref.	-0.03 (-0.26, 0.20)	0.05 (-0.18, 0.29)	0.21 (-0.03, 0.44)

Adjusted for children’s age, sex, primary caregiver, caregiver education, gestational age at birth, birth weight, and duration of breastfeeding.

## Discussion

The prevalence of childhood and adolescent obesity has become a major public health issue in China (25). In addition to being a risk factor for multiple chronic non-communicable diseases in adults, childhood and adolescent obesity can have detrimental effects on various aspects of health, including cardiovascular, endocrine, respiratory, liver, skeletal, psychological, behavioral, and cognitive health (26, 27). In this study, we investigated the relationship between BPA exposure and body composition parameters in children aged 4 to 6 years. Our results indicated that higher BPA levels were associated with higher body composition parameters related to obesity in young children, including BMI, BFM, FFM, and PBF, after adjusting for potential confounding factors.

Our results are in line with prior research that has also reported positive correlations between BPA exposure and obesity-related outcomes in both children and adults. The study conducted by Trasande et al. found that urinary BPA concentrations were positively associated with BMI and AC in children aged 6-19 years (28). Similarly, Carwile et al. reported positive associations between BPA exposure and BMI and waist circumference in adults (29), and Shankar et al. demonstrated positive associations between BPA exposure and BMI and waist circumference in adults aged 18-74 years (30). However, the mechanisms underlying the relationship between BPA exposure and obesity are not fully understood. BPA is a known endocrine disruptor that can alter the body’s hormonal balance and interfere with metabolic pathways, which may contribute to changes in body composition and the development of obesity by moderating inflammation and insulin resistance (31). It has been suggested that BPA may interact with estrogen and G-protein coupled receptors to promote adipogenesis and impair cellular homeostasis (32). BPA is also related to induce epigenetic alterations in specific genes such as Fto, which is involved in appetite control, and alterations at two cis-regulatory elements correlate with transmission of obesity (33). Moreover, BPA exposure has been linked to altered gut microbiota, which may also play a role in the development of obesity and related health issues (34). Further studies are needed to elucidate the underlying mechanisms of the relationship between BPA exposure and obesity-related outcomes in early childhood.

Sex differences in the effects of BPA exposure on obesity-related outcomes have been reported in previous studies, and our study has also showed such differences. Specifically, our results revealed that BPA exposure was more strongly associated with increases in BMI, BFM, PBF, and FMI in males compared to females. However, the results from previous studies have yielded conflicting findings. Liu et al. reported that BPA exposure was more strongly associated with obesity in boys than in girls, despite no significant difference in the median urinary BPA concentrations observed between sexes (35). This suggests that differences in hormone levels may alter the susceptibility to adverse effects related to BPA exposure. However, Gajjar et al. reported conflicting results, did not find compelling evidence that child sex modified the association between urinary BPA concentrations and adiposity outcomes at 8 years of age (36). Additionally, a longitudinal cohort study conducted in California reported that prenatal urinary BPA concentrations were associated with reduced BMI in females at 9 years of age, but not in males (37). In contrast, another study found that elevated BMI levels were observed exclusively in females consuming highly BPA-exposed food (38).

Our study has several strengths, including the use of a well-established method for BPA detection, and the inclusion of a wide range of body composition parameters, which provide a comprehensive picture of the association between BPA exposure and obesity-related outcomes. While our study demonstrated significant positive associations between BPA exposure and body composition parameters in children, with weak effect sizes, it is essential to recognize that even small increases in these parameters may have adverse health consequences over time, particularly in young children whose bodies are still developing. Considering the widespread exposure of BPA in consumer products, our study adds to the growing body of evidence highlighting the need to further examine the health effects of BPA exposure, particularly in vulnerable populations such as infants and children. However, our study is limited by its cross-sectional design, which precludes the establishment of a causal relationship between BPA exposure and changes in body composition parameters. We also recognize that the small sample size may affect the interpretation and generalizability of our findings. Future longitudinal studies with larger sample sizes and more robust study designs are needed to examine the long-term effects of BPA exposure on body composition and obesity-related health outcomes.

## Conclusions

Our study provides further evidence of positive correlations between BPA exposure and body composition parameters in children aged 4 to 6 years. These findings highlight the potential health risks associated with obesity-related body composition parameters in young children. Further investigations are warranted to confirm this association and explore the underlying mechanisms.

## Data availability statement

The raw data supporting the conclusions of this article will be made available by the authors, without undue reservation.

## Ethics statement

The studies involving human participants were reviewed and approved by Medical Research Ethics Board of Guangdong Women and Children Hospital. Written informed consent to participate in this study was provided by the participants’ legal guardian/next of kin.

## Author contributions

Conception and design of this study: YG and J-LW. Data collection and analysis: CL, Y-HD, JN, LY and YG. The first draft of the manuscript was written by YG and all authors commented on previous versions of the manuscript. All authors contributed to the article and approved the submitted version.
