# Supplementary material for: Association between Bisphenol A exposure and body composition parameters in children
Source: Front Endocrinol (Lausanne). 2023 May 18;14:1180505. doi: 10.3389/fendo.2023.1180505 (PMC10234572; doi:10.3389/fendo.2023.1180505)
Supplement: Supplementary file 1 [file DataSheet_1.pdf]

Supplementary Table 1 Correlation between log-transformed BPA levels and body composition parameters

		logBPA	BMI-Z	BFM	SLM	FFM	SMM	PBF	WHR	VFA	AC	AMC	FFMI	FMI	SMI
logBPA	r	1	0.262**	0.260**	0.339**	0.336**	0.343**	0.212**	-0.168*	0.009	0.336**	0.364**	0.290**	0.224**	0.230**
	P-value		<0.001	<0.001	<0.001	<0.001	<0.001	0.003	0.017	0.898	<0.001	<0.001	<0.001	0.001	0.001
BMI-Z	r	0.262**	1	0.915**	0.558**	0.543**	0.558**	0.854**	-0.617**	0.529**	0.958**	0.887**	0.707**	0.933**	0.637**
	P-value	<0.001		<0.001	<0.001	<0.001	<0.001	<0.001	<0.001	<0.001	<0.001	<0.001	<0.001	<0.001	<0.001
BFM	r	0.260**	0.915**	1	0.610**	0.606**	0.607**	0.911**	-0.549**	0.682**	0.925**	0.885**	0.488**	0.969**	0.395**
	P-value	<0.001	<0.001		<0.001	<0.001	<0.001	<0.001	<0.001	<0.001	<0.001	<0.001	<0.001	<0.001	<0.001
SLM	r	0.339**	0.558**	0.610**	1	0.999**	0.999**	0.311**	-0.145*	0.444**	0.712**	0.837**	0.686**	0.434**	0.499**
	P-value	<0.001	<0.001	<0.001		<0.001	<0.001	<0.001	0.04	<0.001	<0.001	<0.001	<0.001	<0.001	<0.001
FFM	r	0.336**	0.543**	0.606**	0.999**	1	0.998**	0.307**	-0.139	0.452**	0.699**	0.827**	0.663**	0.427**	0.464**
	P-value	<0.001	<0.001	<0.001	<0.001		<0.001	<0.001	0.050	<0.001	<0.001	<0.001	<0.001	<0.001	<0.001
SMM	r	0.343**	0.558**	0.607**	0.999**	0.998**	1	0.306**	-0.139*	0.434**	0.713**	0.837**	0.692**	0.431**	0.513**
	P-value	<0.001	<0.001	<0.001	<0.001	<0.001		<0.001	0.05	<0.001	<0.001	<0.001	<0.001	<0.001	<0.001
PBF	r	0.212**	0.854**	0.911**	0.311**	0.307**	0.306**	1	-0.763**	0.447**	0.824**	0.731**	0.282**	0.970**	0.249**
	P-value	0.003	<0.001	<0.001	<0.001	<0.001	<0.001		<0.001	<0.001	<0.001	<0.001	<0.001	<0.001	<0.001
WHR	r	-0.168*	-0.617**	-0.549**	-0.145*	-0.139	-0.139*	-0.763**	1	0.091	-0.601**	-0.524**	-0.249**	-0.659**	-0.261**
	P-value	0.017	<0.001	<0.001	0.04	0.05	0.05	<0.001		0.198	<0.001	<0.001	<0.001	<0.001	<0.001
VFA	r	0.009	0.529**	0.682**	0.444**	0.452**	0.434**	0.447**	0.091	1	0.486**	0.476**	0.253**	0.587**	0.092
	P-value	0.898	<0.001	<0.001	<0.001	<0.001	<0.001	<0.001	0.198		<0.001	<0.001	<0.001	<0.001	0.197
AC	r	0.336**	0.958**	0.925**	0.712**	0.699**	0.713**	0.824**	-0.601**	0.486**	1	0.976**	0.734**	0.899**	0.647**
	P-value	<0.001	<0.001	<0.001	<0.001	<0.001	<0.001	<0.001	<0.001	<0.001		<0.001	<0.001	<0.001	<0.001
AMC	r	0.364**	0.887**	0.885**	0.837**	0.827**	0.837**	0.731**	-0.524**	0.476**	0.976**	1	0.740**	0.815**	0.623**
	P-value	<0.001	<0.001	<0.001	<0.001	<0.001	<0.001	<0.001	<0.001	<0.001	<0.001		<0.001	<0.001	<0.001
FFMI	r	0.290**	0.707**	0.488**	0.686**	0.663**	0.692**	0.282**	-0.249**	0.253**	0.734**	0.740**	1	0.439**	0.893**
	P-value	<0.001	<0.001	<0.001	<0.001	<0.001	<0.001	<0.001	<0.001	<0.001	<0.001	<0.001		<0.001	<0.001
FMI	r	0.224**	0.933**	0.969**	0.434**	0.427**	0.431**	0.970**	-0.659**	0.587**	0.899**	0.815**	0.439**	1	0.388**
	P-value	0.001	<0.001	<0.001	<0.001	<0.001	<0.001	<0.001	<0.001	<0.001	<0.001	<0.001	<0.001		<0.001
SMI	r	0.230**	0.637**	0.395**	0.499**	0.464**	0.513**	0.249**	-0.261**	0.092	0.647**	0.623**	0.893**	0.388**	1
	P-value	0.001	<0.001	<0.001	<0.001	<0.001	<0.001	<0.001	<0.001	0.197	<0.001	<0.001	<0.001	<0.001	

* *P*-value< 0.05, ** *P*-value< 0.01.
